# Impact of different sequential triple oral combination therapies based selexipag on outcomes in pulmonary arterial hypertension

**DOI:** 10.1002/clc.24245

**Published:** 2024-02-25

**Authors:** Xiaoyi Hu, Ping Yuan, Jun Chen, Shang Wang, Hui Zhao, Yaqin Wei, Jiaqi Fu, Fadong Chen, Hongyun Ruan, Wei Zhang, Yanli Zhou, Qiqi Wang, Xiaoling Xu, Kefu Feng, Jianzhou Guo, Sugang Gong, Ruifeng Zhang, Qinhua Zhao, Lan Wang

**Affiliations:** ^1^ Department of Pulmonary Circulation, Shanghai Pulmonary Hospital, School of Medicine Tongji University Shanghai China; ^2^ Department of Cardiology Xiamen Hospital of Traditional Chinese Medicine Fujian China; ^3^ Department of Cardiology, Tongji Hospital Tongji University School of Medicine Shanghai China; ^4^ Department of Cardiology Xuzhou Central Hospital Xuzhou China; ^5^ Department of Rheumatology, Renji Hospital Shanghai Jiaotong University School of Medicine Shanghai China; ^6^ Department of Cardiology First Affiliated Hospital of Nanjing Medical University Nanjing China; ^7^ Department of Cardiology and Atrial Fibrillation Center, The First Affiliated Hospital College of Medicine Zhejiang University Hangzhou Zhejiang China; ^8^ Department of Pulmonary and Critical Care Medicine, Sir Run Run Shaw Hospital Zhejiang University School of Medicine Hangzhou China; ^9^ Department of Cardiology, Division of Life Sciences and Medicine, The First Affiliated Hospital of USTC University of Science and Technology of China Hefei Anhui China; ^10^ Fuwai Hospital Chinese Academy of Medical Sciences Shenzhen Guangdong Province China; ^11^ Department of Respiratory Medicine Zhongda Hospital of Southeast University Nanjing China

**Keywords:** oral sequential triple combination therapy, pulmonary arterial hypertension, selexipag, survival

## Abstract

**Background:**

While the GRIPHON study and others have confirmed the efficacy and safety of selexipag with single, dual, and initial triple combination therapy for patients with pulmonary arterial hypertension (PAH), multicenters studies concerning diverse triple oral combination therapies based on selexipag are limited.

**Hypothesis:**

This study was conducted to evaluate the effects of various sequential triple oral combination therapies on PAH outcomes.

**Methods:**

A retrospective study was carried out involving 192 patients from 10 centers, who were receiving sequential triple oral combination therapy consisting of an endothelin receptor antagonist (ERA), a phosphodiesterase 5 inhibitor (PDE5i)/riociguat and selexipag. Clinical parameters, event‐free survival, and all‐cause survival were assessed and analyzed at baseline and posttreatment.

**Results:**

Among the 192 patients, 37 were treated with ERA + riociguat + selexipag, and 155 patients received ERA + PDE5i + selexipag. Both sequential triple oral combination therapies improved the World Health Organization functional class and raised the count of low‐risk parameters. As a result of the larger patients' population in the ERA + PDE5i + selexipag group, these individuals exhibited significant increases in 6‐minute walking distance (6MWD), pulmonary arterial systolic pressure, pulmonary arterial pressure, right ventricle, and eccentricity index, and significant decreases in N‐terminal probrain natriuretic peptide after 6 months of treatment. Nevertheless, both sequential triple oral combination therapy groups demonstrated similar shifts in these clinical parameters between baseline and 6 months. Baseline 6MWD and mean pulmonary arterial pressure were independent predictors of survival in patients undergoing ERA + PDE5i + selexipag therapy. Importantly, no significant differences were found in 6‐month event‐free survival and all‐cause survival between two groups.

**Conclusions:**

Different oral sequential triple combination therapies based on selexipag could comparably improve outcomes in patients with PAH.

## Graphical abstract

Among the 192 pulmonary arterial hypertension patients, 37 were treated with endothelin receptor antagonist (ERA) + riociguat + selexipag, and 155 patients received ERA + phosphodiesterase 5 inhibitor + selexipag. Patients of various ages and genders were likely to be prescribed one of these six triple sequential oral combination therapies.

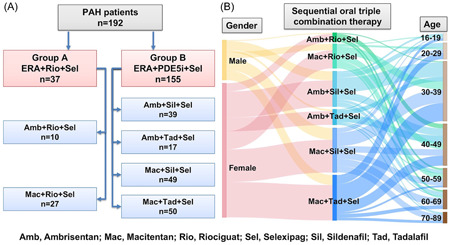

**Abbreviations**
6MWD: 6‐minute walking distance
AP: mean right atrial pressure
AWP: mean pulmonary arterial wedge pressure
BMI: body mass index
CHD‐PAH: congenital heart disease‐associated pulmonary arterial hypertension
CI: cardiac index
CO: cardiac output
CTD‐PAH: connective tissue disease‐associated pulmonary arterial hypertension
DBP: diastolic blood pressure
EI: eccentricity index
ERA: endothelin receptor antagonist
HHT: hereditary hemorrhagic telangiectasia
HR: heart rate
IPAH: idiopathic pulmonary arterial hypertension
LVEDD: left ventricular end‐diastolic diameter
LVEF: left ventricular ejection fraction
mPAP: mean pulmonary arterial pressure
NT‐proBNP: N‐terminal probrain natriuretic peptide
PASP: pulmonary arterial systolic pressure
PDE5i: phosphodiesterase 5 inhibitor
PE: pericardial effusion
PVR: pulmonary vascular resistance
RA area: right atrial area
RAP: right atrial pressure
RR: respiratory rate
RV: right ventricle
SBP: systolic blood pressure
Sm: mitral annular peak systolic velocity
SVO2: mixed venous oxygen saturation
TAPSE: tricuspid annular plane systolic excursion
TPR: total pulmonary resistance
TR: tricuspid regurgitation
WHO FC: World Health Organization functional class

## 1 INTRODUCTION

Pulmonary arterial hypertension (PAH) is a deadly, progressively worsening vascular disease with a poor prognosis.^1^ The incidence and prevalence of PAH are estimated at up to 2.4 cases and 15 cases per million annually, respectively.^2^, ^3^ Abnormally high mean pulmonary arterial pressure (mPAP) and pulmonary vascular resistance (PVR) are consequences of pathological alterations in various proliferative and inflammatory signaling pathways of pulmonary vascular cells.^4^, ^5^ Currently, PAH medication primarily targets the vasoploriferative and vasoconstrictive pathways in pulmonary vasculature. Conventional drugs include prostacyclin analogs, endothelin receptor antagonists (ERAs), phosphodiesterase 5 inhibitors (PDE5is), and soluble guanylate cyclase stimulators. These treatments have significantly improved the survival rate of PAH patients over the last two decades, achieving a 7‐year survival rate of 50%.^6^, ^7^

With the advances in target therapy, triple combination therapy targeting nitric oxide, prostacyclin, and endothelin‐1 pathways has also garnered recommendations. Selexipag, an orally available, selective prostacyclin receptor agonist, when used alone or in conjunction with mono or double therapy involving an ERA and/or a PDE5i, reduces the relative risk of composite morbidity/mortality events by 40%.^8^ However, the 2022 ESC/ERS guidelines for the diagnosis and treatment of PH discourage the use of initial tripple therapy with macitentan and tadalafil for PAH patients at low and intermediate‐low risk.^9^ For patients at intermediate‐high or high risk where intravenous/subcutaneous prostacyclin analogs may not be feasible, oral sequential selexipag‐based triple combination therapy may be an option. Despite studies analyzing the effectiveness and safety of initial selexipag‐based triple combination therapy for patients with PAH,^8^, ^9^, ^10^, ^11^ data on the impact of different triple sequential oral combination therapies based on selexipag on outcomes of patients with PAH remains limited.

Therefore, our study aims to analyze the distinct impacts of two triple sequential oral combination therapy‐ ERA + riociguat + selexipag and ERA + PDE5i + selexipag, on short‐ and long‐term outcomes in patients with PAH.

## 2 METHODS

### 2.1 Study population and design

This multicenter retrospective study included 192 patients (aged 16–80 years) diagnosed with PAH at Shanghai Pulmonary Hospital and nine other hospitals in China between January 2019 and June 2022. The inclusion criteria entailed a mPAP of at least 25 mmHg, a pulmonary arterial wedge pressure (PAWP) of 15 mmHg or less, and a PVR exceeding 3 Wood units. These patients had been on a dual‐combination therapy with ERA and either PDE5i or riociguat for a minimum of 3 months but still exhibited disease progression or unsatisfactory long‐term clinical response. Consequently, they underwent a sequential addition of selexipag. The starting dosage of selexipag was 200 µg twice daily, and it was recommended to incrementally increase the dosage by 200 µg twice daily every week, contingent on the patient's tolerance to adverse effects. The maximum tolerated dose reached up to 1600 µg twice daily. Exclusion criteria included patients with severe cardiopulmonary complications, uncorrected congenital heart disease, or portal hypertension. The study adhered to the Declaration of Helsinki and received approval from the Ethics Committee of Shanghai Pulmonary Hospital (number: L21‐222). Written informed consent was secured from all patients.

### 2.2 The assessment of clinical parameters

The initial evaluation incorporated demographic data, 6‐minute walking distance (6MWD), levels of N‐terminal probrain natriuretic peptide (NT‐proBNP), World Health Organization functional class (WHO FC), risk assessment, hemodynamics and echocardiography parameters.

The hemodynamics parameters, obtained through right cardiac catheterization, included the mean right atrial pressure (RAP), mPAP, PAWP, cardiac output, cardiac index, PVR, total pulmonary resistance, and mixed venous oxygen saturation.

In accordance with the guidelines established by the American Society of Echocardiography, we evaluated several echocardiographic parameters. These included pulmonary arterial systolic pressure (PASP), tricuspid regurgitation, right atrial area, RAP, right ventricle (RV), mitral annular peak systolic velocity, left ventricular end‐diastolic diameter (LVEDD), left ventricular ejection fraction, eccentricity index (EI), tricuspid annular plane systolic excursion (TAPSE), and presence of pericardial effusion (PE).

### 2.3 The assessment of outcomes

During the follow‐up period, events and survival were collected. The events included hospitalization due to heart failure, the discontinuation of selexipag by patients, and all‐cause death. In cases where multiple events occurred for a single patient, only the initial event was taken into account for analysis. We also conducted a detailed analysis of the 6‐month event‐free survival and all‐cause survival.

### 2.4 The follow‐up protocol

The patients were monitored either through out‐patient clinic visits or via telephonic check‐ins every 3 months. After 6‐month of treatment, all clinical parameters, excluding hemodynamic ones, were reassessed and recorded for every patient. For individuals who lost to follow‐up, we confirmed their survival status as of May 23, 2023.

### 2.5 Statistical analysis

All data were presented as the mean ± standard deviation and categorical data were presented as counts or percentages. Normal distribution was evaluated using the Kolmogorov–Smirnov test. Clinical variables comparisons at baseline and after 6 months of treatment were made using one‐way analysis of variance for normally distributed variables and the nonparametric Friedman test for variables not following a normal distribution. The Chi‐square test was used to identify differences in categorical variables between baseline and the 6‐month mark. Differences in clinical parameters across various groups were scrutinized using the Wilcoxon signed‐rank test. The impact of parameters on prognosis was assessed using univariate Cox proportional hazards analyses. Variables that reached a significance level of *p* < .05 were included in multivariable Cox analyses. To estimate event‐free survival and all‐cause survival, we employed the Kaplan–Meier method. Statistical significance was set at *p* < .05. All data were stored in a spreadsheet on personal computer‐based data. Analysis and graphing were conducted using the Statistical Package for the Social Sciences software (version 21.0), GraphPad Prism (version 9), and Dishu Tubiao (https://dycharts.com).

## 3 RESULTS

### 3.1 Baseline clinical characteristics of patients

The baseline clinical characteristics of the patients are detailed in Table 1. Out of the 192 PAH patients, 37 received a combination of ERA, riociguat, and selexipag, while 155 patients were treated with a combination of ERA, PDE5i, and selexipag. Female patients constituted 78.4% and 76.8% of these groups, respectively. No significant differences were found between the two groups in terms of demographic data, classification, WHO FC, 6MWD, NT‐proBNP, risk stratifications, event, adverse event and hemodynamics characteristics.

**Table 1:** Baseline clinical characteristics.

Characteristics	ERA + riociguat + selexipag (*n* = 37)	ERA + PDE5i + selexipag (*n* = 155)	*p* Value
Age, years	43.8 ± 11.9	42.8 ± 15.4	.711
Female, *n* (%)	29 (78.4)	119 (76.8)	.835
BMI,	23.7 ± 4.0	22.4 ± 4.4	.306
HR, bpm	86.8 ± 12.7	81.6 ± 18.6	.122
RR, bpm	20.1 ± 1.5	19.7 ± 1.8	.265
SBP, mmHg	108.3 ± 12.2	112.8 ± 14.8	.099
DBP, mmHg	66.4 ± 9.9	69.1 ± 12.8	.248
Classification			.362
IPAH, *n* (%)	23 (62.2)	74 (47.7)	
CHD‐PAH, *n* (%)	6 (16.2)	40 (25.8)	
CTD‐PAH, *n* (%)	8 (21.6)	38 (24.5)	
HHT, *n* (%)	0 (0)	3 (1.9)	
WHO FC II/III/IV, *n*	6/24/7	34/102/19	.484
6MWD, m	271.8 ± 173.4	313.4 ± 146.9	.210
NT‐proBNP, pg/mL	2041(1218–3870)	1445 (475–3016)	.126
Risk stratifications, *n* (%)			
1/2/3/4^a^	4/2/17/14	18/33/74/30	.035
Event, *n* (%)	6 (16.2)	17 (11.0)	.440
Adverse event,^b^ *n* (%)	16 (43.2)	66 (42.6)	.963
Hemodynamics characteristics			
mRAP, mmHg	12.5 ± 11.7	7.4 ± 6.0	.120
mPAP, mmHg	72.0 ± 10.8	62.0 ± 17.2	.112
mPAWP, mmHg	13.5 ± 6.5	9.3 ± 3.8	.072
CO, L/min	4.3 ± 1.6	4.2 ± 1.3	.766
CI, L/min/m^2^	2.5 ± 0.7	2.5 + 0.8	.965
PVR, WU	13.4 ± 7.2	13.9 ± 6.8	.817
TPR, WU	14.9 ± 10.7	11.6 ± 8.9	.426
SVO_2_, %	58.2 ± 4.8	61.5 ± 11.2	.391

*Note*: Data are presented as *n* (%), mean ± standard deviation, and interquartile range.

Abbreviations: 6MWD, 6‐minute walking distance; BMI, body mass index; CHD‐PAH, congenital heart disease‐associated pulmonary arterial hypertension; CI, cardiac index; CO, cardiac output; CTD‐PAH, connective tissue disease‐associated pulmonary arterial hypertension; DBP, diastolic blood pressure; ERA, endothelin receptor antagonist; HHT, hereditary hemorrhagic telangiectasia; HR, heart rate; IPAH, idiopathic pulmonary arterial hypertension; mPAP, mean pulmonary arterial pressure; mPAWP, mean pulmonary arterial wedge pressure; mRAP, mean right atrial pressure; NT‐proBNP, N‐terminal probrain natriuretic peptide; PDE5i, phosphodiesterase 5 inhibitor; PVR, pulmonary vascular resistance; RR, respiratory rate; SBP, systolic blood pressure; SVO2, mixed venous oxygen saturation; TPR, total pulmonary resistance; WHO FC, World Health Organization functional class; WU, wood units.

^a^ 1/2/3/4, low/intermediate‐low//intermediate‐high/high risk.

^b^ Means the 6 months‐long‐term adverse event not the short‐term adverse effect after taking medication.

**Figure 1:**
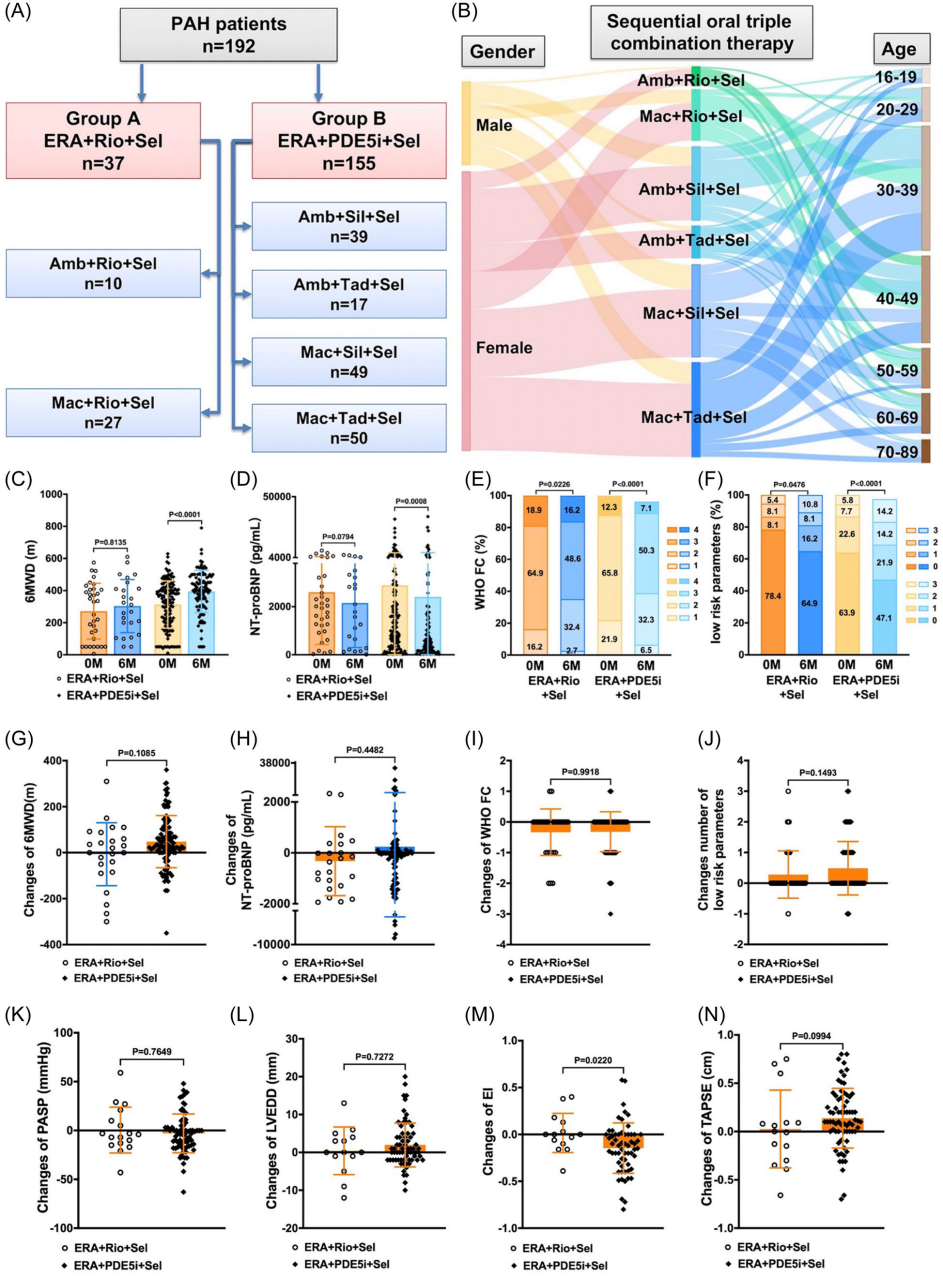
(A and B) The details of sequential oral triple combination therapy. (C–J) The differences and changed several clinical parameters of patients treated with 6 months between the two oral tiple combination therapies. (K–N) Changes in several echocardiographic parameters of patients treated for 6 months between the two oral tiple combination therapies. Amb, ambrisentan; ERA, endothelin receptor antagonist; Mac, macitentan; PDE5i, phosphodiesterase 5 inhibitor; Rio, riociguat; Sel, selexipag; Sil, sildenafil; Tad, tadalafil.

In the group with ERA, riociguat, and selexipag, 10 patients received a combination of ambrisentan, riociguat, and selexipag, while 27 were treated with macitentan, riociguat, and selexipag. In contrast, while the ERA, PDE5i, and selexipag group consisted of 39 patients with ambrisentan, sildenafil, and selexipag; 17 patients with ambrisentan, tadalafil, and selexipag; 49 patients with macitentan, sildenafil, and selexipag; and 50 patients with macitentan, tadalafil, and selexipag (Figure 1A). Patients of various ages and genders were likely to be prescribed one of these six triple sequential oral combination therapies (Figure 1B).

### 3.2 Changes in several clinical parameters between baseline and 6 months

The changes in several clinical parameters after 6 months of oral triple combination therapy were depicted in Figure 1C–J. Notably, improvement was observed in the 6MWD, NT‐proBNP, WHO FC, and number of reaching low risk parameters after 6 months of the therapy (Figure 1C–F). These parameters also showed enhancement in patients treated with a combination of ERA, PDE5i, and selexipag. However, in the group receiving ERA, riociguat, and selexipag, only the WHO FC and number of low‐risk parameters showed improvement. Furthermore, there were no significant differences in the changes of 6MWD, NT‐proBNP, WHO FC, and number of low‐risk parameters between patients receiving ERA + PDE5i + selexipag and those treated with ERA + riociguat + selexipag for 6 months (Figure 1G–J).

### 3.3 The comparison of echocardiographic characteristics between baseline and 6 months

The echocardiographic characteristics at baseline and 6 months are displayed in Table 2. With the larger sample size of patients treated with ERA + PDE5i + selexipag, improvements in cardiac remodeling were observed in after 6 months of therapy, as evidence by the enhancement in the PASP, RAP, RV, LVEDD, EI, TAPSE, and PE. Furthermore, there were no discernible changes in the echocardiographic characteristics of patients after 6 months of treatment with ERA, riociguat, and selexipag. However, notable improvements were seen in PASP, RAP, RV, LVEDD, EI, TAPSE, and PE for patients following 6 months of treatment with ERA + PDE5i + selexipag.

**Table 2:** Baseline and 6 months echocardiographic characteristics.

	ERA + riociguat + selexipag (*n* = 37)		ERA + PDE5i + selexipag (*n* = 155)	
Characteristics	0 month	6 months	0 month	6 months
PASP, mmHg	95.8 ± 23.8	96.8 ± 25.5	92.0 ± 32.1	86.6 ± 30.8*
TR	4.8 ± 0.3	5.2 ± 0.5	4.8 ± 0.9	4.5 ± 0.7
RA area, cm^2^	29.0 ± 11.3	27.8 ± 13.9	28.3 ± 15.6	27.0 ± 16.6
RAP, mmHg	16.3 ± 17.2	9.8 ± 6.0	11.2 ± 7.9	9.2 ± 4.8*
RV, cm	4.9 ± 0.9	5.1 ± 1.3	5.2 ± 1.4	5.0 ± 1.4*
Sm, cm/s	10.4 ± 3.5	10.9 ± 1.9	10.5 ± 2.6	10.7 ± 2.4
LVEDD, mm	33.6 ± 7.4	34.1 ± 5.5	35.5 ± 7.1	37.4 ± 6.7**
LVEF (%)	78.3 ± 5.5	88.7 ± 6.1	80.1 ± 6.4	78.0 ± 7.9
EI	1.6 ± 0.4	1.7 ± 0.4	1.9 ± 0.7	1.8 ± 0.6**
TAPSE, cm	1.6 ± 0.4	1.7 ± 0.4	1.6 ± 0.4	1.7 ± 0.7**
PE, *n*(%)	16 (51.6)	7 (46.7)	49 (36.3)	15 (19.0)*

*Note*: Data are presented as *n* (%), mean ± standard deviation, and interquartile range.

Abbreviations: EI, eccentricity index; ERA, endothelin receptor antagonist; LVEDD, left ventricular end‐diastolic diameter; LVEF, left ventricular ejection fraction; PASP, pulmonary arterial systolic pressure; PDE5i, phosphodiesterase 5 inhibitor; PE, pericardial effusion; RA right atrial area; RAP, right atrial pressure; RV, right ventricle; Sm, mitral annular peak systolic velocity; TAPSE, tricuspid annular plane systolic excursion; TR, tricuspid regurgitation.

* *p* < .05 versus 0 month.

** *p *< .01 versus 0 month.

The alterations in various echocardiographic parameters of patients who underwent 6 months of oral tiple combination therapy were presented in Figure 1K–N. Notably, the change in EI was less pronounced in patients treated with ERA, PDE5i, and selexipag compared to those who received ERA, riociguat, and selexipag. However, no significant differences were observed in the changes of PASP, LVEDD, and TAPSE between patients treated with ERA + PDE5i + selexipag and those with ERA + riociguat + selexipag over 6‐month period.

### 3.4 The Cox proportional hazards analysis of outcomes

**Figure 2:**
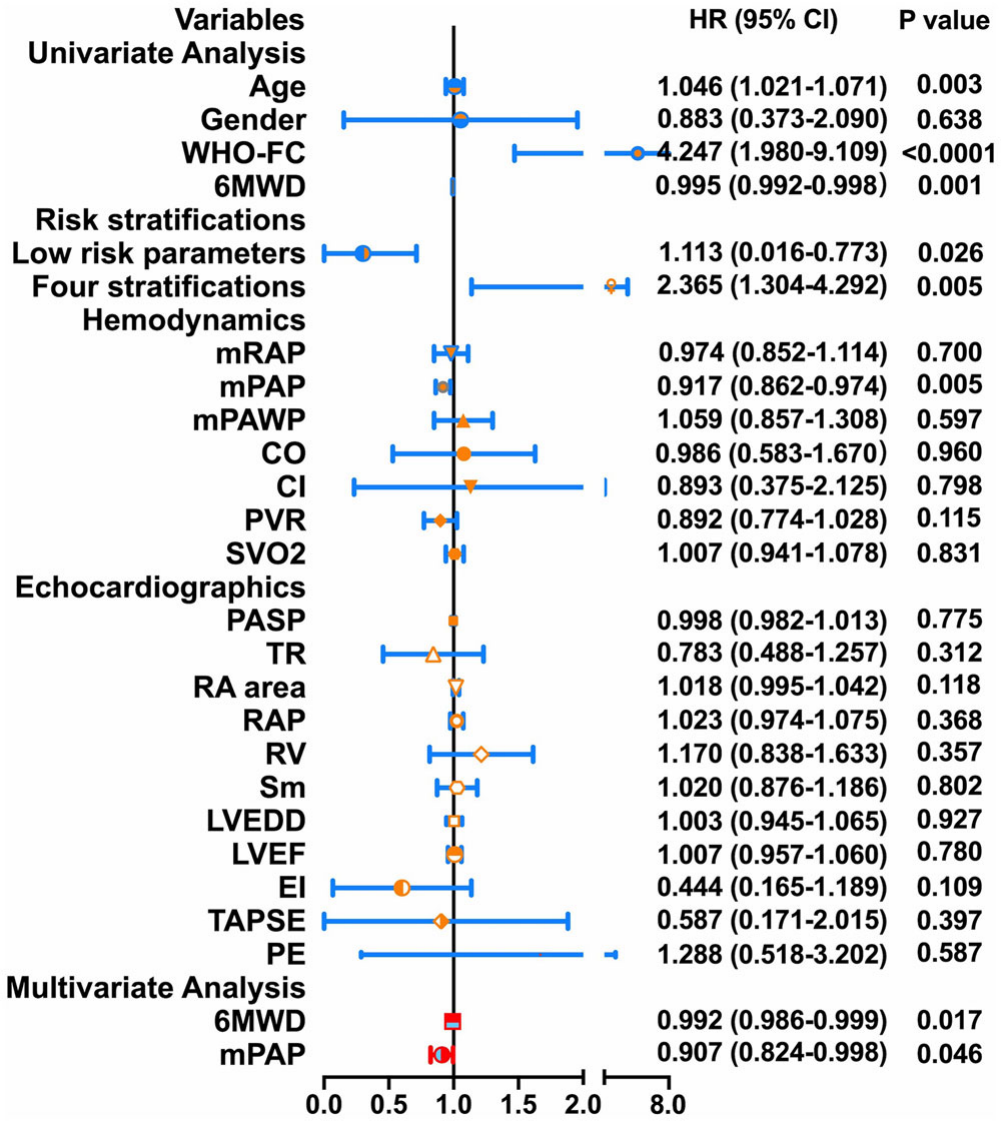
The cox regression analysis between the clinical parameters and survival in patients treated with endothelin receptor antagonist + phosphodiesterase 5 inhibitor + selexipag.

Adequate sample size is critical for regression analysis. Therefore, we examined the parameters influencing the survival of patients treated with ERA + PDE5i + selexipag. In the univariate Cox proportional hazards analysis, factors such as age, WHO FC 6MWD, risk stratifications, and mPAP were associated with survival in these patients. Moreover, in the multivariate forward stepwise Cox proportional hazards analysis, 6MWD and mPAP levels emerged as independent predictors of survival in patients treated with ERA + PDE5i + selexipag (Figure 2).

### 3.5 6‐month event‐free survival and all‐cause survival analysis

**Figure 3:**
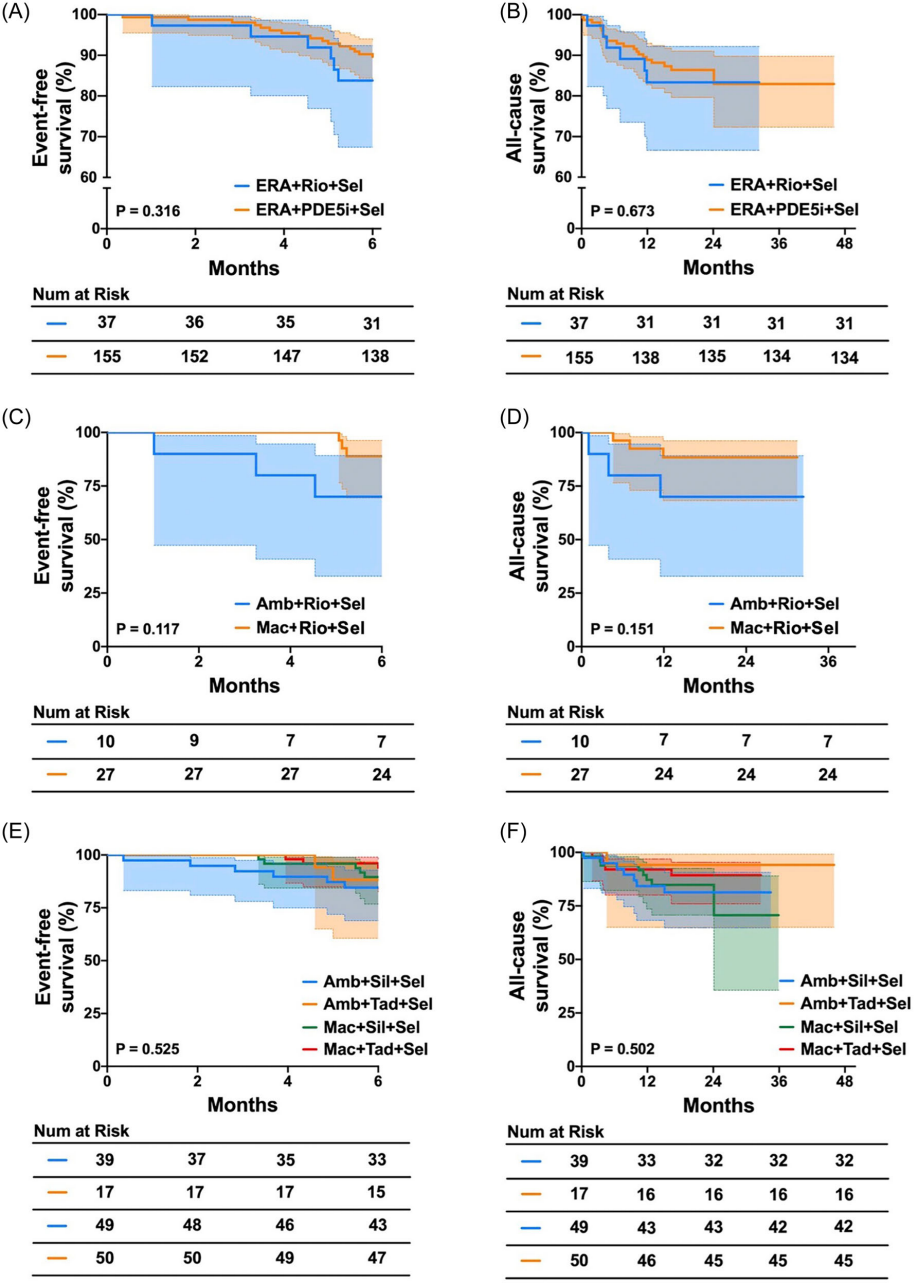
(A) The event‐free survival for patients treated with ERA+Rio+Sel and ERA+PDE5i+Sel. (B) The all‐cause survival for patients treated with ERA+Rio+Sel and ERA+PDE5i+Sel. (C) The event‐free survival for patients treated with Amb+Rio+Sel and Mac+Rio+Sel. (D) The all‐cause survival for patients treated with Amb+Rio+Sel and Mac+Rio+Sel. (E) The event‐free survival for patients treated with Amb+Sil+Sel, Amb+Tad+Sel, Mac+Sil+Sel, and Mac+Tad+Sel. (F) The all‐cause survival for patients treated with Amb+Sil+Sel, Amb+Tad+Sel, Mac+Sil+Sel, and Mac+Tad+Sel. Amb, ambrisentan; ERA, endothelin receptor antagonist; Mac, macitentan; PDE5i, phosphodiesterase 5 inhibitor; Rio, riociguat; Sel, selexipag; Sil, sildenafil; Tad, tadalafil.

We conducted an analysis of the 6‐month event‐free survival and all‐cause survival in patients treated with oral triple combination therapy (Figure 3). There results indicated no significant differences in both 6‐month event‐free survival and all‐cause survival between patients treated with ERA + riociguat + selexipag and ERA + PDE5i + selexipag (Figure 3A,B). Similarly, no significant differences were observed in the 6‐month event‐free survival and all‐cause survival between two subgroups treated with ambrisentan + riociguat + selexipag and macitentan + riociguat + selexipag, as well as among the subgroups with ERA + PDE5i + selexipag. These subgroups included 39 patients with ambrisentan + sildenafil + selexipag, 17 patients with ambrisentan + tadalafil + selexipag, 49 patients with macitentan + sildenafil + selexipag, and 50 patients with macitentan + tadalafil + selexipag (Figure 3C–F).

The estimated 6‐month event rates of patients with ERA + riociguat + selexipag and ERA + PDE5i + selexipag were 16% and 11%, respectively (Figure 3A). Furthermore, the estimated 1‐year survival rates of patients with ERA + riociguat + selexipag and ERA + PDE5i + selexipag were 84% and 89%, respectively (Figure 3B).

## 4 DISCUSSION

In this study, we found that different oral sequential triple combination therapies based on selexipag could comparably improve outcomes in patients with PAH. Notably, exercise capacity, NT‐proBNP level, and right ventricular functions showed significantly improvement in patients treated with ERA + PDE5i + selexipag after 6 months. And 6MWD and mPAP were the independent predictor of survival in patients treated with ERA + PDE5i + selexipag. Although similar shifts in clinical parameters between baseline and 6 months were shown in both sequential triple oral combination therapy groups, no statistical differences were shown in patients treated with ERA + riociguat + selexipag group. This may due to the fact that the sample size of the ERA + riociguat + selexipag group was much smaller, or different ERA was used. Numerous studies have demonstrated that, compared to monotherapy, both sequential dual therapy and initial dual therapy can significantly improve hemodynamic parameters and right heart function, decrease the incidence of clinical events, and extend survival in patients with PAH. The initial combination therapy consisting an ERA and a PDE5i is recommended for patients presenting with low or intermediate risk and is the most frequently used clinical strategy.^9^ However, some patients still experience limited efficacy of dual therapy, and their long‐term survival rate is less than promising.^8^, ^10^ Therefore, patients with PH at intermediate‐high or high risk may derive benefit from initiating or transitioning to triple combination therapy.

The GRIPHON study demonstrated that selexipag is the first nonprostanoid agonist of the prostacyclin receptor that can reduce the risk of PAH disease progression, lower the morbidity/mortality composite endpoint, and delay PAH progression.^11^, ^12^, ^13^, ^14^ Both the GRIPHON and TRITON clinical trials showed that the initiation of selexipag treatment within 6 months reduced the risk of disease progression for PAH patients who were already receiving dual background therapy with ERA and PDE5i.^15^ A recent study also suggested that the ERA (macitentan) + riociguat + selexipag effectively improved clinical parameters and was well‐tolerated in patients with PAH who had low/intermediate risk, and possibly even in half of high‐risk patients.^16^ These findings align with our results, which indicate that both ERA + PDE5i + selexipag and ERA + riociguat + selexipag enhanced cardiopulmonary function. The smaller sample size of patients treated with ERA + riociguat + selexipag led to less statistical differences in clinical parameters. However, when compared to the group treated with ERA + riociguat + selexipag, the changes in clinical parameters from baseline to 6 months were similar in the group with ERA + PDE5i + selexipag, as were the 6‐month event‐free survival and all‐cause survival. This suggests that PAH patients treated with either ERA + PDE5i + selexipag or ERA + riociguat + selexipag may have comparable short‐ and long‐term prognosis, enabling PAH patients to choose either style of oral sequential triple combination therapy according to their specific circumstances.

In our study, we found that 1‐year survival rates were 84% for patients treated with ERA + riociguat + selexipag and 89% for those treated with ERA + PDE5i + selexipag. These findings align with the 2022 ESC/ERS Guidelines for the diagnosis and treatment of pulmonary hypertension.^9^ However, Cui et al. reported a higher 1‐year event‐free survival rate of 96.7% in PAH patients who received selexipag‐based initial triple combination therapy.^17^ The difference in our data may be due to variations in gender ratios, risk stratification, and the type of initial triple or sequential triple combination therapy. Nevertheless, previous studies and our own indicated that selexipag‐based initial triple combination therapy has a positive impact on the prognosis of PAH patients.^14^, ^16^, ^17^

Our study had several limitations. First, this was a retrospective multicenter study with a relatively small selected patients' group rather than a prospective multicenter study. Second, the hospital cases included were primarily concentrated in East China, which may not accurately reflect trends across the whole of China. Third, most of the patients were female and in the middle to older age range, potentially underrepresenting teenagers and young patients. Furthermore, the sample size of ERA + riociguat + selexipag group is much smaller than that in the ERA + PDE5i + selexipag group. Finally, the follow‐up period was relatively short and we did not collect catheterization data during this period.

## 5 CONCLUSIONS

Our data demonstrated that various selexipag‐based oral sequential triple combination therapies, namely ERA + riociguat + selexipag and ERA + PDE5i + selexipag, could similarly improve outcomes for patients with PAH. Therefore, different selexipag‐based sequential oral triple therapy combinations could be successfully implemented. Physicians can choose an appropriate selexipag‐based combination therapy according to the patient's specific physical and economic conditions.

## AUTHOR CONTRIBUTIONS

Xiaoyi Hu, Ping Yuan, and Lan Wang were directly involved in the patients' recruitment and care, contributed to the study design, study conduct and supervision, scientifific overview, data analysis, and editing of the manuscript. Xiaoyi Hu, Ping Yuan, Jun Chen, Fadong Chen, Hongyun Ruan, Yanli Zhou, Qiqi Wang, Xiaoling Xu, Kefu FengF, Jianzhou Guo, Sugang Gong, and Ruifeng Zhang contributed to patient enrollment, data analysis, scientific interpretation, drafting, and editing the original manuscript. Wei Zhang contributed to data collection and curation, and formal analysis. All authors have reviewed the manuscript and approved the final version for submission.

## CONFLICT OF INTEREST STATEMENT

The authors declare no conflict of interest.

## Data Availability

The raw data supporting the conclusions of this article will be made available by the corresponding authors without undue reservation.
